# Supplementary material for: Relationship between the honeydew of mealy bugs and the growth of Phlebopus portentosus
Source: PLoS One. 2020 Jun 12;15(6):e0233710. doi: 10.1371/journal.pone.0233710 (PMC7292412; doi:10.1371/journal.pone.0233710)
Supplement: Attachment — Submitted filename: Comments to Author.docx [file pone.0233710.s003.docx]

**Comments to the Author**

The manuscript (PONE-D-19-30898) entitled “Relationships between the honeydew of mealy bugs and the growth of *Phlebopus portentosus*” has been processed. The results suggest that honeydew secreted by mealy bugs can attract and improve the mycelial growth of *P. portentosus* and form a fungal-insect gall. It will be useful for understand how *P. portentosus* benefit from this symbiotic relationship. The detailed comments and suggestions are as follows.

1. In the whole passage, “fungus-insect” should be changed to “fungal-insect”.

2. Line 30, “agar medium” but not “agar media”.

3. Line 36, 56 and 199, “sugar” should be changed to “sugars”.

4. Line 40, “fungi” should be changed to “fungus”.

5. Line 72-73, the singular and plural should be consistent in this sentence.

6. Line 95, “was” should be deleted.

7. Line 102, “by” should be deleted.

8. Line 214, “a fungi” rather than “a fungus”.

9. Whole numbers less than ‘10’ should be written as words rather than numbers, such as line 147-148. In return, when numbers more than ‘10’ should be written as numbers rather than word, such as line 140.

10. The language should be further improved.
